# Evaluation of D1 and D2 Dopamine Receptor Segregation in the Developing Striatum Using BAC Transgenic Mice

**DOI:** 10.1371/journal.pone.0067219

**Published:** 2013-07-02

**Authors:** Dominic Thibault, Fabien Loustalot, Guillaume M. Fortin, Marie-Josée Bourque, Louis-Éric Trudeau

**Affiliations:** Departments of Pharmacology and Neuroscience, Neuroscience Research Group, Faculty of Medicine, Université de Montréal, Montréal, Québec, Canada; Prince Henry's Institute, Australia

## Abstract

The striatum is predominantly composed of medium spiny neurons (MSNs) that send their axons along two parallel pathways known as the direct and indirect pathways. MSNs from the direct pathway express high levels of D1 dopamine receptors, while MSNs from the indirect pathway express high levels of D2 dopamine receptors. There has been much debate over the extent of colocalization of these two major dopamine receptors in MSNs of adult animals. In addition, the ontogeny of the segregation process has never been investigated. In this paper, we crossed bacterial artificial chromosome *drd1a-tdTomato* and *drd2-GFP* reporter transgenic mice to characterize these models and estimate D1-D2 co-expression in the developing striatum as well as in striatal primary cultures. We show that segregation is already extensive at E18 and that the degree of co-expression further decreases at P0 and P14. Finally, we also demonstrate that cultured MSNs maintain their very high degree of D1-D2 reporter protein segregation, thus validating them as a relevant *in vitro* model.

## Introduction

The striatum is the input nucleus of the basal ganglia, a neuronal network crucial for action selection and motor control [1]–[3]. The vast majority of neurons that form the striatum are GABAergic projection neurons called medium spiny neurons (MSNs). It is well accepted that MSNs send their axons in two parallel and mostly exclusive pathways: either to the external segment of the globus pallidus via the indirect pathway, or to the substantia nigra pars reticulata and the internal segment of the globus pallidus via the direct pathway [4]–[7]. As their name implies, MSNs express a high density of dendritic spines with which afferent glutamatergic fibers from the cortex and the thalamus form excitatory synapses [8]. MSNs also receive important inputs from dopaminergic (DAergic) neurons of the substantia nigra pars compacta [9]–[12]. Although the extent and relevance of co-expression of D1 and D2 receptors in MSNs is still the subject of considerable debate [13], [14], MSNs that form the direct pathway have consistently been found to express high amounts of D1 dopamine (DA) receptors and very little D2 DA receptors. Conversely, MSNs of the indirect pathway express high amounts of D2 DA receptors and very little D1 DA receptors [14]–[22]. Much of the available data on D1/D2 co-expression in MSNs has been obtained in mature animals, leaving the establishment of the DA receptor segregation through development mostly unexplored [7]. In addition, although MSN neurons in primary culture are a commonly used *in vitro* model, whether D1/D2 segregation is faithfully maintained in culture is unclear. For example, some groups have reported very high colocalization of D1 and D2 receptors *in vitro* with either binding assays [23] or immunolabeling [14], [24]–[26], suggesting a loss of segregation *in vitro* [7]. Due to limitations in the sensitivity and selectivity of the techniques used to detect DA receptors in these previous studies, an examination of D1 and D2 gene expression using approaches such as transgenic reporter mice is warranted and could greatly clarify the issue.

Transgenic animals expressing reporter genes that encode fluorescent proteins, represent invaluable tools in the investigation of individual MSN populations [27]–[29]. In the present study, we crossed *drd1a*-tdTomato and *drd2*-GFP bacterial artificial chromosomes (BAC) transgenic mice to examine D1 and D2 reporter gene expression both *in vivo* and *in vitro*. We found that *in vivo*, at embryonic day 18 (E18), at birth (P0) and at postnatal day 14 (P14), there is never more than about 10% colocalization between the D1 and D2 reporter genes. Furthermore, we find a gradual decrease in the percentage of colocalization from E18 to P0 and P14, reaching levels under 5%. Additionally, we demonstrate that this segregation is maintained *in vitro*, independently of the identity of neuronal populations interacting with MSNs (mesencephalic and/or cortical neurons).

## Materials and Methods

### Animals

BAC transgenic *drd1a*-tdTomato [30] and *drd2*-GFP [31] mice, backcrossed on a C57BL/6 background, were maintained in individual colonies. Hemizygous animals from each strain were crossed to yield double-transgenic animals at the expected ratio of 25% per litter. For C57BL/6 animals (WT) were used in single-cell RT-PCR experiments. All experiments were approved by the Université de Montréal animal ethics committee (CDEA) (protocol #11–191). All efforts were made to minimize the number of animals used and their suffering.

### Brain Slice Preparation

The rationale for selecting E18, P0 and P14 was as follows: P0 and P14 were used in order to establish a general comparison with primary cultures, which were obtained from P0 mouse brains and kept 2 weeks in culture before use; E18 was the earliest time point at which it was still possible to reliably dissect the dorsal striatum. For embryonic animals, gestating mice were anesthetized with halothane and quickly decapitated. Embryos were extracted and put in ice-cold physiological saline solution containing (in mM): 140 NaCl, 5 KCl, 2 MgCl_2_, 2 CaCl_2_, 10 HEPES, 6 sucrose and 10 glucose, adjusted at pH 7.35. P0 pups were cryoanesthetized, and P14 pups were anesthetized with halothane. All brains were quickly harvested upon anesthesia and maintained in ice-cold saline solution for assessment of genotype under a fluorescence microscope; only pups showing both red (tdTomato) and green (GFP) fluorescence were selected for experiments. Double-transgenic brains were immediately fixed in 4% PFA for 48 hours at 4°C. Coronal sections (100 µm thick) were cut in ice-cold PBS with a Leica VT1000s vibrating microtome (Leica Microsystems, Wetzlar, Germany). Sections (8 per brain) at the middle of the rostro-caudal axis of the dorsal striatum were selected for quantification.

### Acutely Dissociated Neurons

The dissociations were performed according to a previously described protocol [32]. Briefly, a block of dorsal striatum was manually dissected from double-transgenic brains that were harvested in the same manner as for slice preparation. For E18 and P0 brains, the striatal tissue block was incubated for 20 min at 37°C in papain (Worthington Biochemicals) and then mechanically dissociated into a cell suspension. Striatal blocks from P14 brains were incubated for 30 min at 30°C with trypsin in an oxygenated piperazine-N-N-bis(2-ethane sulfonic acid) (PIPES) solution containing (in mM): 120 NaCl, 2 KCl, 0.5 CaCl2, 1 MgCl2, 25 glucose, and 20 PIPES at pH 7.0. These blocks were then washed 2 times with an oxygenated PIPES solution containing 10% fetal bovine serum, then left to rest at room temperature in an oxygenated PIPES solution for 1 h before subsequent mechanical dissociation into a cell suspension. All striatal cell suspensions (E18, P0 and P14) were seeded on polyethyleneimine (PEI)-coated glass coverslips at a density ranging from 250 000 to 500 000 cells/mL, depending on yield, and maintained at room temperature for 15 min before fixation for 30 min in 4% PFA.

### Postnatal Primary Neuron Cultures

Cultures were prepared according to a previously described protocol [33]. Four different types of cultures were prepared. Briefly, dissociated neurons from the dorsal striatum (double-transgenic) and/or cortex (wild-type) and/or ventral mesencephalon (wild-type) of P0-P2 animals were seeded on a monolayer of cortical astrocytes (wild-type) grown on poly-L-lysine-coated glass coverslips. The total seeded neuron density was always 240,000 cells/mL broken down as follows (in cells/mL): 240,000 striatal cells for monocultures (Mono); 140,000 striatal cells plus 100,000 cortical cells for co-cortical cultures (CoCx); 140,000 striatal cells plus 100,000 mesencephalic cells for co-mesencephalic cultures (CoMs); 40,000 striatal cells plus 100,000 mesencephalic cells, plus 100,000 cortical cells for triple cultures (3x). The objective of using such ratios was to maintain a constant proportion of cortical and mesencephalic neurons between CoCx, CoMs and 3x, such that the influence of each population on DA receptor expression by MSNs would be relatively similar; the amount of striatal cells was therefore adjusted accordingly to maintain the same total cell density. These cultures, incubated at 37°C in 5% CO_2_, were maintained in Neurobasal medium enriched with 1% penicillin/streptomycin, 1% Glutamax, 2% B-27 supplement and 5% fetal bovine serum.

### Immunostaining and Image Quantification

**Figure 1:**
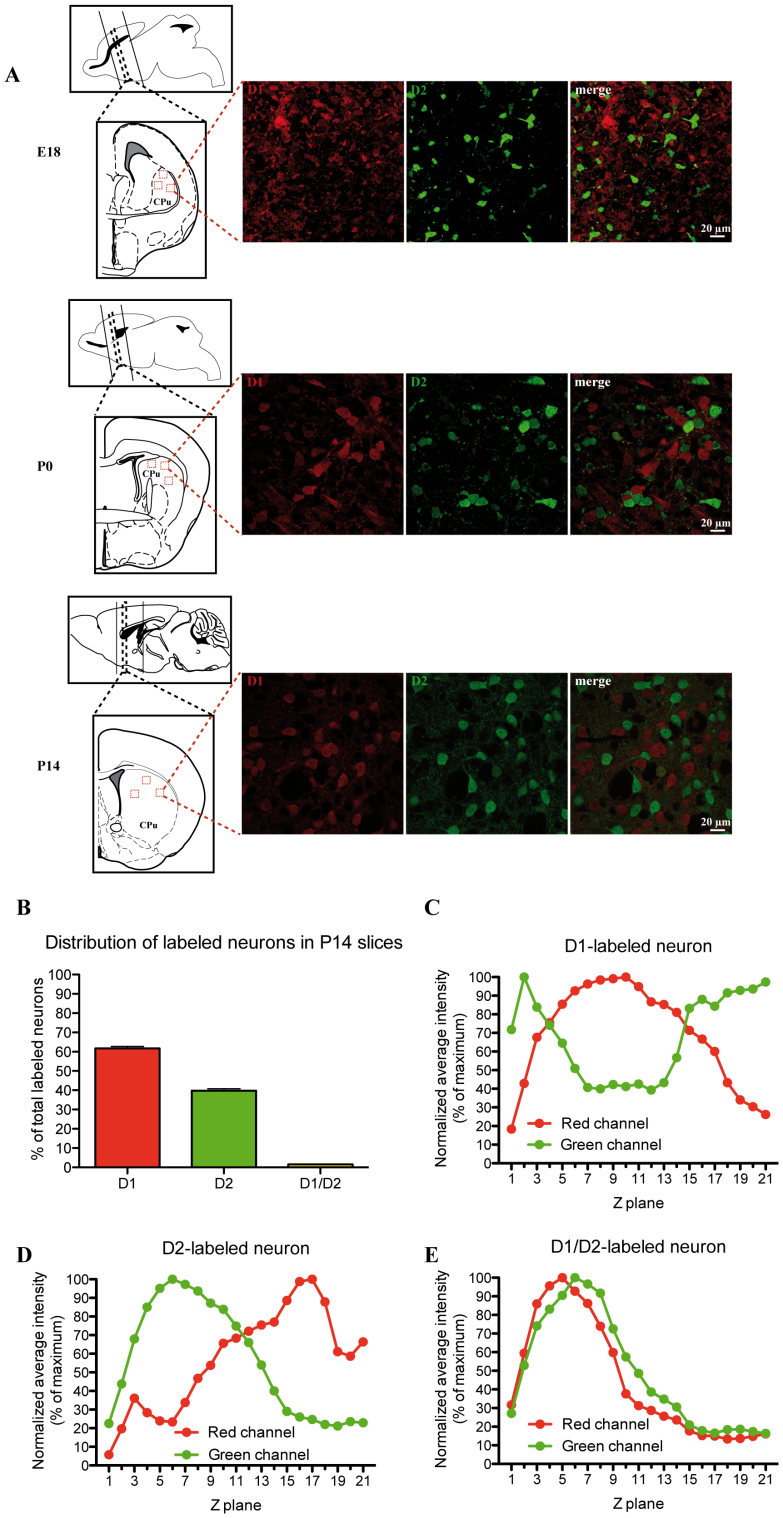
Distribution of D1 and D2 reporter proteins in mouse striatal sections reveals extensive segregation. Characterization of tdTomato (D1, red) and GFP (D2, green) immunolabeling in striatal sections prepared from E18, P0 and P14 double-transgenic mice. **A**: The brain atlas images [44], [45] on the left identify the approximate areas where acute slices were obtained. The confocal images shown on the right were obtained using a 60x objective. Although the tdTomato (D1, red) signal was relatively diffuse in E18 and P0 sections, at P14, immunopositive cell bodies could be easily identified. Double-labelled neurons were not frequently detected, as shown in the merge images on the right. **B**: Summary diagram presenting the results of quantifications performed in striatal sections from P14 animals showing the proportion of total labeled MSNs that express tdTomato (D1), GFP (D2) or both proteins (D1/D2). **C**–E: Orthogonal distribution of normalized average intensities from single typical D1- (C), D2- (D) and D1/D2-labeled (E) neurons.

Brain sections, acutely dissociated neurons and primary cultured neurons were immunolabeled for tdTomato (D1-MSNs) and GFP (D2-MSNs) with the following antibodies: rabbit anti-RFP (Rockland, Gilbertsville, PA, USA, 1∶500) detected with ALEXA 546 goat anti-rabbit (Molecular Probes, Eugene, OR, USA, 1∶500) and chicken anti-GFP (Millipore, Billerica, MA, USA, 1∶1000) detected with ALEXA 488 goat anti-chicken (Molecular Probes, 1∶500). Images were captured with a laser scanning confocal microscope (FV1000 MPE, Olympus) equipped with multi-argon and helium/neon lasers. For brain sections (P14 only), 6 microscopic fields of the dorsal striatum (3 per hemisphere, see Figure 1) were averaged together to obtain a single value (n) of each parameter (D1-labelled, D2-labelled, D1/D2-labelled) per section. Similarly, 5 and 6 images per coverslips of acutely dissociated or cultured neurons were averaged together (2 separate experiments for acutely dissociated, 4 for cultured neurons) to obtain each individual value (n). All quantifications were performed blindly by first evaluating neurons in individual detection channels to assess the distribution of D1 and D2 MSN populations, followed by merging the single images to identify and count doubly-labeled neurons. Double-labeled neurons are thus included in the D1 and D2 MSN counts. The values shown always represent a percentage of the total number of counted fluorescent neurons, as only D1- and/or D2-labeled cells were counted. To obtain the normalized intensity distribution curves of orthogonal planes (Fig. 1C–E), a straight line (width = 1 pixel) was first drawn across one typical neuron of each phenotype. For each channel, the intensity of all pixels in the line were then averaged together, normalized with the maximal intensity value of that channel and plotted for all planes of the Z-series.

### Single-cell RT-PCR

Collection of acutely dissociated neurons was performed as previously described [34] from the dorsal part of the dorsal striatum of P0 and P14 WT mice. Half of the cDNA was used to amplify D1 mRNA. The other half was used to amplify D2S (short isoform) and/or D2L (long isoform) mRNA. cDNA synthesis was performed as previously described [34]. A first round of PCR was carried out by using half of the RT reaction and 30 cycles at 55°C of annealing temperature. A second round of PCR was performed using 10% of the first PCR and 31 cycles at 55°C of annealing temperature. Primers were synthesized by AlphaDNA (Montréal, Québec). Nested PCRs were performed during the second round of amplification. The identity of PCR products was confirmed by sequencing. Primers were as follows: D1∶5′- cctgttttctgtccctgctta -3′ and 5′- ccatgtaggttttgccttgtg -3′; D1 nested (232 bp): 5′- tctttgtcatctctttagctgtgtc -3′ and 5′- ttcggagtcatcttcctctca -3′; D2∶5′- attgtctgggtcctgtccttc -3′ and 5′- atctgagtggctttcttctcctt -3′; D2 nested (D2l:417 bp, D2s: 330 bp): 5′- tactcctccatcgtctcgttcta -3′ and 5′- atgcccattcttttctggttt -3′. PCRs were resolved in 1.5% agarose gels.

### Statistical Analysis

Data are presented as mean ± SEM. The level of statistical significance was established at *p*<0.05 in one-way and two-way ANOVAs. Statistical outliers were excluded when they differed by more than two standard deviations above or below the mean (one P14 brain slice, two coverslips of acutely dissociated neurons and two coverslips of neuronal cultures were thus excluded from final analyses).

## Results

### Quantification of Labeled Neurons in Striatal Sections

We first characterized tdTomato (D1-positive neurons) and GFP (D2-positive neurons) immunostaining in coronal brain sections of double-transgenic mice at E18, P0 and P14. In sections from P14 mice (Fig. 1A), 61.70±1.03% of dorsal striatal neurons were D1-positive and 39.81±1.01% were D2-positive (Fig. 1B, n = 7). Only a very small proportion of those were D1/D2-positive (1.51±0.22%). In brain sections prepared from E18 and P0 mice, the tdTomato signal to noise ratio in neuronal cell bodies was typically too low to obtain reliable quantifications of the proportion of immunopositive neurons (Fig. 1A). To validate our counting method in P14 slices, we qualitatively compared the orthogonal distribution of the average signal intensity across typical D1-, D2 and D1/D1-labeled neuron. Neurons positive for only one reporter protein (Fig. 1C and 1D) display little overlap in the normalized signal intensity distribution, while a doubly-labeled neuron displays a very similar peak and overall distribution, arguing for a high degree of colocalization (Fig. 1E).

### Quantification of Labeled Neurons after Acute Dissociation

**Figure 2:**
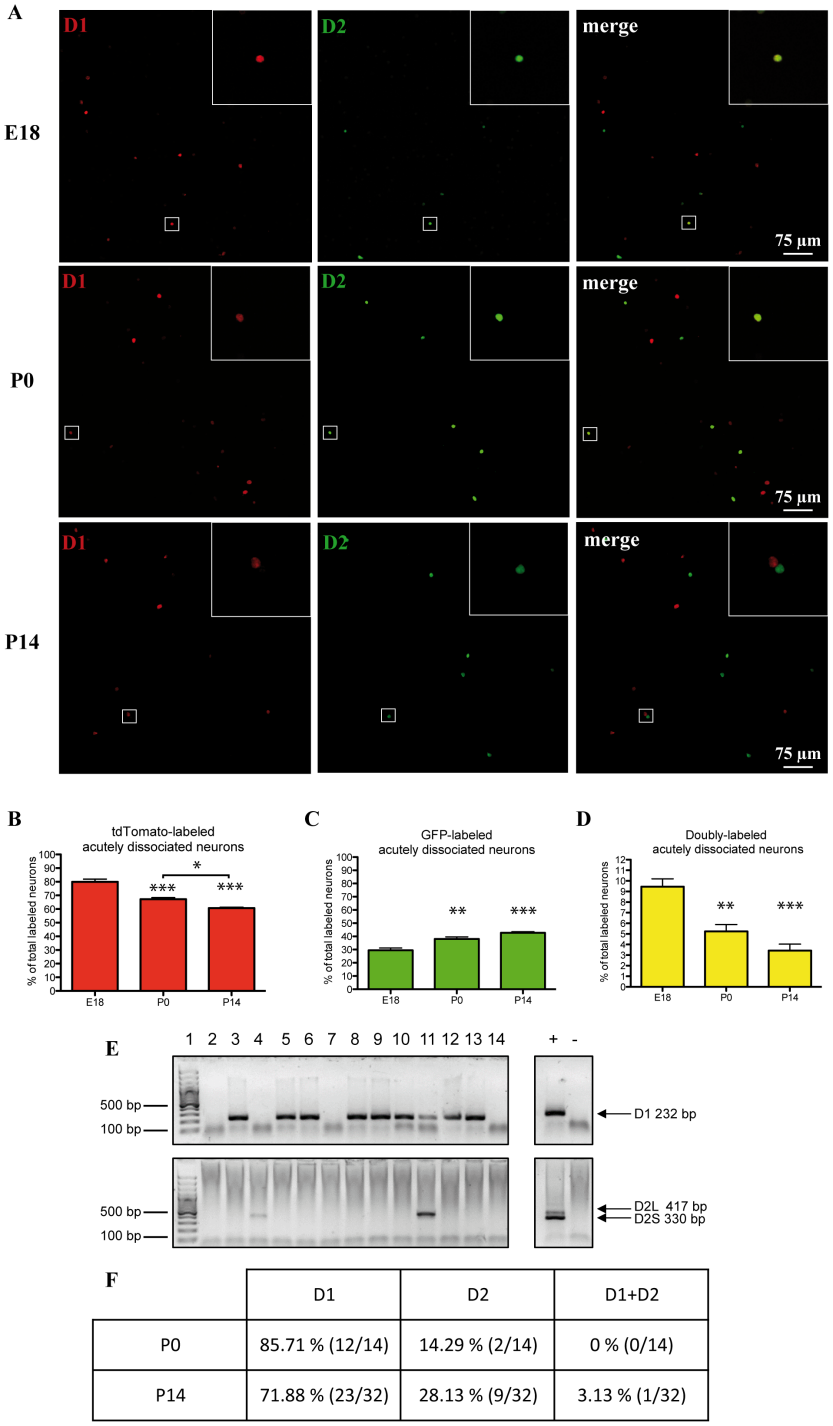
Distribution of D1 and D2 reporter proteins in acutely dissociated mouse striatal sections reveals an age-dependent increase in segregation. Characterization of tdTomato (D1, red) and GFP (D2, green) immunolabeling in neurons acutely dissociated from E18, P0 and P14 striatum from double-transgenic mice. **A**: Examples of acutely dissociated striatal neurons immunolabeled for tdTomato (D1, red) and GFP (D2, green). **B–D**: Summary diagrams presenting the results of quantifications performed from E18, P0 and P14 mice **(**tdTomato in B, GFP in C and both proteins in D). **E**: Single-cell RT-PCR from freshly dissociated P14 WT striatal cells. In this example (wells 2–14), nine collected neurons expressed D1 receptor mRNA and two collected neurons expressed D2L receptor mRNA. One neuron collected expressed both receptors mRNA (well 11). Positive control: whole mesencephalon RNA; negative control: water. **F**: Table summarizing the results of single-cell RT-PCR experiments performed with P0 and P14 WT mice.

We immunostained acutely dissociated striatal neurons to further examine D1 and D2 reporter gene expression at earlier developmental time points. We found that in acutely dissociated P14 MSNs, 60.73±0.66% of dorsal striatal neurons were D1-positive and 42.70±0.85% were D2-positive (Fig. 2). Only 3.43±0.61% of those were D1/D2-positive. Although not statistically significant, there was a tendency for a more efficient detection of D1/D2-expressing MSNs in acutely dissociated neurons in comparison to brain sections (*p* = 0.053 for slices versus acutely dissociated, two-way ANOVA, n = 7), most likely because of the better signal to noise ratio. Extending this analysis to P0 and E18 tissue, we observed a significant decrease of the proportion of D1-expressing neurons as a function of age (79.99±1.92% at E18, 67.20±1.21% at P0 and 60.73±0.66% at P14, *p*<0.001, one-way ANOVA, Fig. 2B). The opposite effect was observed for D2-expressing MSNs, with the proportion increasing as a function of age (29.46±1.76% at E18, 38.03±1.59% at P0 and 42.70±0.85% at P14, *p*<0.001, one-way ANOVA, Fig. 2C). The proportion of D1-expressing MSNs at E18 was significantly higher than at P0 and P14, and P0 was also higher than P14, while the proportion of D2-expressing MSNs at E18 was significantly lower than at P0 and P14 (*p*<0.001 for D1 at E18 versus P0 and P14, *p*<0.05 for D1 at P0 versus P14, *p*<0.01 for D2 at E18 versus P0 and P14, Bonferroni's multiple comparison test, n = 8 for E18, 15 for P0 and 7 for P14). We also observed a decrease in the percentage of doubly-labeled D1/D2-expressing neurons at P0 and P14 compared to E18 (9.45±0.75% at E18, 5.24±0.65% at P0 and 3.43±0.61% at P14; *p*<0.001 in one-way ANOVA; *p*<0.001 for E18 versus P0 and P14 in Bonferroni's multiple comparison test, n = 8 for E18, 15 for P0 and 7 for P14, Fig. 2D). Overall, our results indicate that there are more neurons that express D1 than D2 reporter constructs in double-transgenic mice at each time points examined, with a more important difference at the earliest time point (E18). Moreover, we find that the level of D1/D2 co-expression is already relatively low at E18 and continues to decrease during early postnatal development.

**Figure 3:**
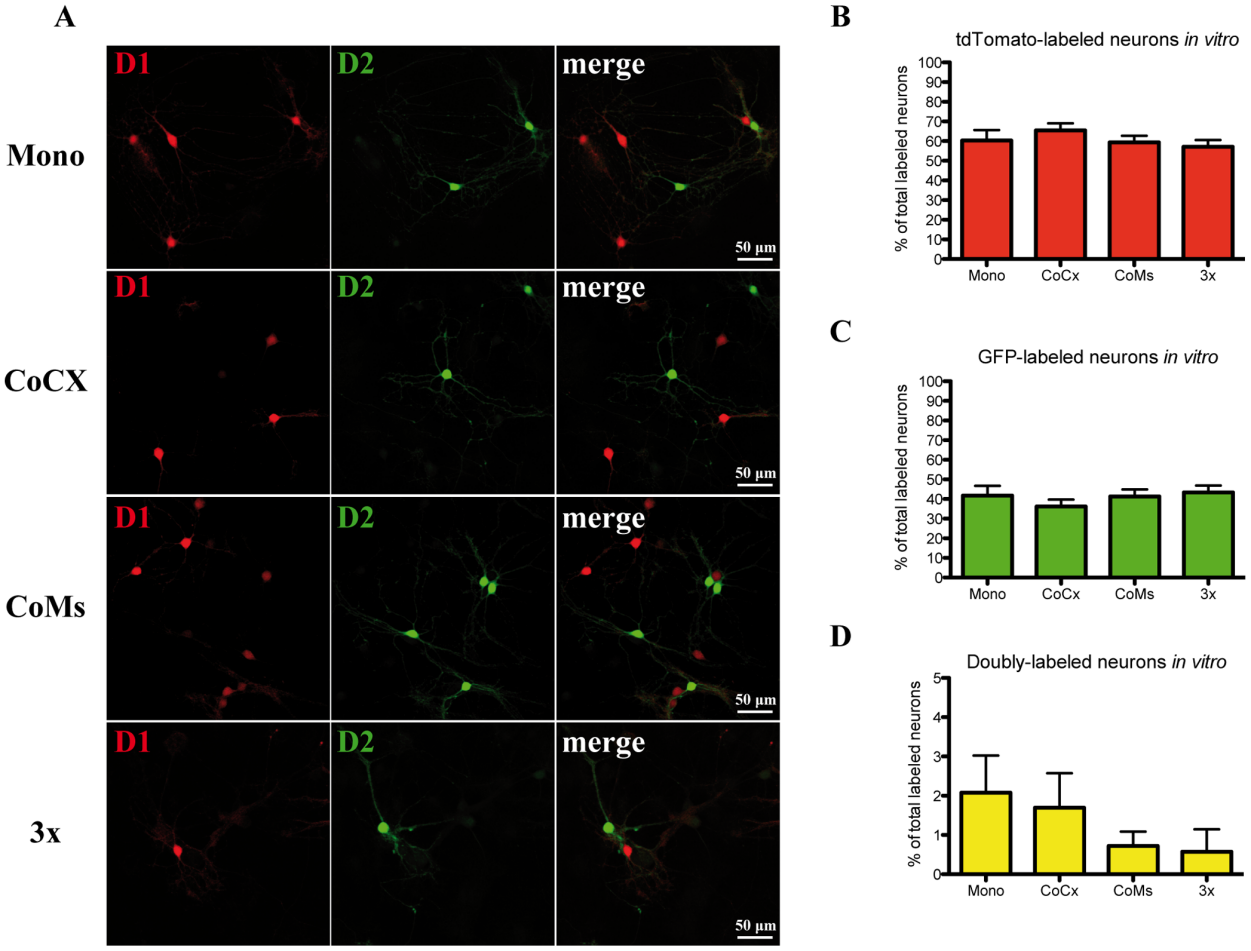
Segregation of D1 and D2 reporter proteins is maintained in postnatal striatal neurons in primary culture. Four types of culture conditions were compared: striatal neurons alone (**Mono**), striatal neurons cultured with cortical neurons (**CoCx**), striatal neurons cultured with mesencephalic neurons (**CoMs**), or mixed cultures containing striatal neurons, mesencephalic neurons and cortical neurons (**3x**). **A**: Examples of MSNs in different culture conditions labeled for tdTomato (D1, red) and GFP (D2, green) at 14 days *in vitro*. **B–D**: Summary diagrams showing the results of quantifications. **B**: tdTomato, **C**: GFP, **D**: both proteins.

To validate our data obtained from reporter gene analysis, we performed single-cell RT-PCR detection of D1 and D2 mRNA in acutely dissociated striatal neurons obtained from P0 and P14 WT mice. Although the proportions of D1 and D2 expressing neurons were not identical with the two approaches, single-cell RT-PCR analysis confirmed the three main conclusions reached by evaluating reporter gene expression, namely that (1) there was a much larger proportion of D1-positive compared to D2-positive neurons (D2L) at both ages, (2) there was a decrease in D1-positive and increase in D2-positive neurons from P0 to P14 (85.71% D1- and 14.29% D2-positive neurons at P0, 71.88% D1- and 28.13% D2-positive neurons at P14, n = 14 for P0 and 32 for P14, Fig. 2E–F) and (3) neurons expressing both mRNA were very infrequent (none detected out of 14 neurons at P0 and 1/32 neurons at P14, Fig. 2F).

### Quantification of Labeled Neurons in Culture

The preservation of D1-D2 segregation *in vitro* has been previously questioned on many accounts [14], [23]–[26], [35]. To resolve this controversy, we next examined reporter gene expression in primary cultured MSNs prepared from P0 double-transgenic mice. In order to determine if D1-D2 segregation was further influenced by neuronal interactions, we compared four different culture conditions: striatal neurons alone (Mono), striatal neurons with cortical neurons (CoCx), striatal neurons with mesencephalic neurons (CoMs) or striatal neurons with mesencephalic and cortical neurons (3x). Neurons were fixed at 14 days *in vitro* (DIV) and processed for tdTomato and GFP immunocytochemistry to count neurons that expressed either D1- or D2-driven fluorescent reporter proteins (Fig. 3A).

First, we observed that there was no significant difference in the percentage of tdTomato (D1)- and GFP (D2)-expressing neurons in the different culture conditions (D1: *p* = 0.56, Fig. 3B; D2: *p* = 0.63, Fig. 3C; one-way ANOVA, n = 7–8 culture coverslips per group). In fact, the approximate 60∶40 ratio of tDTomato (D1)- and GFP (D2)-expressing neurons seen in brain sections and in acutely dissociated neurons at P14 was fully maintained between different culture conditions *in vitro* (Fig. 3). Second, the extent of fluorescent reporter colocalization in cultured neurons was very limited across culture types *in vitro* (Fig. 3D), with values similar to those observed in acutely dissociated neurons at P14 (Fig. 2D). Although a tendency for reduced coexpression in cultures containing mesencephalic dopamine neurons was observed, statistical analysis showed that there was no significant effect of the culture type (one-way ANOVA; *p* = 0.40, n = 7–8 culture coverslips per group). These results show that the segregation of D1 and D2 gene expression is essentially preserved *in vitro*.

## Discussion

In this study, we characterized D1 and D2 reporter gene expression in striatal neurons of *drd1a*-tdTomato/*drd2*-GFP double-transgenic mice of different ages and in several striatal culture conditions to obtain an estimation of D1-D2 DA receptor coexpression. Our first main result is that there is a globally higher proportion of neurons expressing the D1 than the D2 gene at all time points examined. Second, we found a decrease in the percentage of purely D1-expressing neurons to the profit of an increase of purely D2-expressing neurons in the striatum of P0 and P14 compared to E18 animals. Our third main result is that the shift from D1 to D2 reporter gene expression is accompanied by a decrease in the percentage of D1/D2 doubly-labeled neurons. Finally, we show that D1/D2 reporter protein segregation is fully maintained in postnatal MSNs in primary culture, with colocalization of the fluorescent markers remaining below 5%.

Over the years, there has been much debate over the exact degree of segregation between the direct and indirect projection pathways of MSNs, particularly concerning their selective expression of DA receptor subtypes [15]–[21]. Although striatal neurons expressing both receptors exist, leading to D1-D2 heteroreceptor formation with novel pharmacological properties [14], [22], the general consensus that has gradually emerged is that in the adult animal, most direct pathway striatonigral MSNs express high levels of D1 receptor while most indirect pathway striatopallidal MSNs express high levels of D2 receptor [7]. Studies performed since the introduction of BAC *drd2*-GFP and *drd1a*-tdTomato transgenic mice have confirmed the high degree of D1-D2 dopamine receptor segregation in striatal neurons [30], [36]. However, the initial establishment of this segregation during development had never been described before the present study.

In the present work, based on the analysis of reporter genes driven by DA receptor promoters, we reproduce previous data showing a general segregation between D1 and D2 receptor expression in the striatum. Our experimental samples always contained the entire striatal neuronal population, including around 90–95% of MSNs and 5–10% of various interneurons subpopulations, all of which express significant amounts of DA receptors [10]. We did not attempt to examine separately the small contingent of GABAergic or cholinergic interneurons; considering the small proportion of non-MSN neurons, the results presented here should represent a fair assessment of the MSN population, despite the obvious possibility of a modest error. We demonstrate that there is a higher percentage of MSNs labeled with tdTomato than with GFP, and that the difference is greater at E18 than P0 or P14. We also show the same basic tendency by single-cell RT-PCR measurements of D1 and D2 mRNA in acutely dissociated striatal neurons from P0 and P14 WT mice. These results may appear to be in contradiction with a previous report showing that D2 receptor mRNA is more abundant than D1 receptor mRNA in the developing mouse striatum [37]. However, it should be emphasized that our analysis was restricted to the *percentage of cells* that expressed D1- and D2-driven reporter proteins or D1 and D2 mRNA, while the actual global mRNA and protein levels were not quantified. Interestingly, we noted that purely GFP-positive neurons at E18 generally exhibited much stronger fluorescence signal intensity than that seen in tdTomato-positive neurons (results not shown), suggesting that at early time points, although there are less D2-expressing neurons, those that express the D2 receptor could do so at a higher level than the D1 receptor in D1-positive neurons. Our data also show that the decrease in the percentage of D1-positive neurons was accompanied by a gradual increase in D2-positive neurons and a decrease in D1/D2 fluorescent reporter protein colocalization from E18 to P0 and P14. Taken together, these results suggest the possibility that newly differentiated MSNs might express mostly the D1 receptor early on in development, until some of them start expressing gradually more of the D2 receptor and less of the D1, to eventually become purely D2 over time. An investigation of neurons prior to E18 would be useful to further evaluate this hypothesis.

What signals drive and maintain the differentiation of D1- and D2-expressing MSNs is presently undetermined. Many non-mutually exclusive possibilities should be considered, including the existence of an intrinsic genetic program, the production of local signals from cell populations inherent to the striatum or afferent innervation, or signals deriving from the establishment of MSN efferent projections to various target regions. Given the very early stage of segregation onset observed in the present study, the implication of intrinsic or local factors appears likely, but the presence of DAergic terminals in the striatum during embryonic development [38]–[40] and the fact that the DA release machinery is already functional at birth [41] certainly raise the possibility that DA could be involved in the segregation mechanism. However, our observation that cultured MSNs, whether in monoculture or in mixed cultures with mesencephalic and/or cortical neurons, maintain D1-D2 segregation, is compatible with the hypothesis that DA receptor expression pattern is intrinsic to MSNs or at least becomes hardwired soon after its onset. Importantly, although our findings concerning DA receptor segregation in culture are in agreement with other similar but qualitative observations made using the same double transgenic model [42], they are in apparent contradiction with other studies showing colocalization of D1 and D2 receptors in a range spanning from 22 to 90% of cultured striatal neurons from either embryonic or postnatal rats [14], [23]–[26], [35]. In particular, a recently published paper demonstrated a stable DA receptor phenotype in rat striatal neurons through 4, 11 and 25 days in culture, with approximately 90% of D1-D2 colocalization [26]. Two main explanations can be considered for such differences. A first possibility is that this contradiction results from a species difference: perhaps rat striatal neurons show less receptor segregation than mouse striatal neurons. Arguing against this possibility, previous in situ hybridization studies performed in rat provided strong evidence for extensive segregation of D1 and D2 receptor mRNA in separate cell populations [15], [20], [43]. Therefore, it is to be expected that neuronal cultures derived from rat striatum should also show extensive segregation. A second possibility, perhaps more likely, is that the higher rate of coexpression found in the studies performed in rat cultures may have resulted from a high rate of false positive signal caused by the limited specificity of the classical immunocytochemistry or fluorophore-labeled receptor antagonist techniques used. In the present study, the distinction between cell bodies positive or negative for the reporter proteins was relatively straightforward and was facilitated by the accumulation of the fluorescent reporter proteins inside neuronal cell bodies. Because of our counting strategy that involved the use of an intensity threshold, it is possible that colocalization occurring in neurons that express relatively low levels of the reporter proteins may have been missed. However, our single-cell RT-PCR data argue against such a possibility. The high level of similarity between the results obtained in brain sections, acutely dissociated and cultured neurons, analyzed using the very same approach, argues in favor of an overall maintenance of DA receptor segregation in vitro.

Lastly, the comparison between D1 and D2 reporter expression across four different types of postnatal cultures revealed a small tendency for reduced coexpression in cultures containing mesencephalic dopamine neurons. Although this did not reach statistical significance, this observation suggests a possible influence of DAergic neurons on the process of segregation. Further experiments will be required to evaluate this hypothesis, but our results make it clear that the presence of DAergic or cortical neurons is not required for the general maintenance of DA receptor expression pattern after birth.

## References

[1] Mink JW (1996) The basal ganglia: focused selection and inhibition of competing motor programs. Prog Neurobiol 50: 381–425. 9004351 10.1016/s0301-0082(96)00042-1

[2] Redgrave P, Prescott TJ, Gurney K (1999) The basal ganglia: a vertebrate solution to the selection problem? Neuroscience 89: 1009–1023. 10362291 10.1016/s0306-4522(98)00319-4

[3] Cisek P, Kalaska JF (2010) Neural mechanisms for interacting with a world full of action choices. Annu Rev Neurosci 33: 269–298 doi:10.1146/annurev.neuro.051508.135409 20345247 10.1146/annurev.neuro.051508.135409

[4] Kawaguchi Y (1997) Neostriatal cell subtypes and their functional roles. Neurosci Res 27: 1–8. 9089693 10.1016/s0168-0102(96)01134-0

[5] Smith Y, Bevan MD, Shink E, Bolam JP (1998) Microcircuitry of the direct and indirect pathways of the basal ganglia. Neuroscience 86: 353–387. 9881853 10.1016/s0306-4522(98)00004-9

[6] Wu Y, Richard S, Parent A (2000) The organization of the striatal output system: a single-cell juxtacellular labeling study in the rat. Neurosci Res 38: 49–62. 10997578 10.1016/s0168-0102(00)00140-1

[7] Bertran-Gonzalez J, Hervé D, Girault J-A, Valjent E (2010) What is the Degree of Segregation between Striatonigral and Striatopallidal Projections? Front Neuroanat 4. Available: http://www.ncbi.nlm.nih.gov/pubmed/20953289. Accessed 2012 Feb 9. 10.3389/fnana.2010.00136 PMC2955397 20953289

[8] Bolam JP, Hanley JJ, Booth PA, Bevan MD (2000) Synaptic organisation of the basal ganglia. J Anat 196 (Pt 4): 527–542. 10.1046/j.1469-7580.2000.19640527.x PMC1468095 10923985

[9] Nicola SM, Surmeier J, Malenka RC (2000) Dopaminergic modulation of neuronal excitability in the striatum and nucleus accumbens. Annu Rev Neurosci 23: 185–215 doi:10.1146/annurev.neuro.23.1.185 10845063 10.1146/annurev.neuro.23.1.185

[10] Gerfen CR, Surmeier DJ (2011) Modulation of striatal projection systems by dopamine. Annu Rev Neurosci 34: 441–466 doi:–10.1146/annurev-neuro-061010–113641 21469956 10.1146/annurev-neuro-061010-113641 PMC3487690

[11] Rice ME, Patel JC, Cragg SJ (2011) Dopamine release in the basal ganglia. Neuroscience 198: 112–137 doi:10.1016/j.neuroscience.2011.08.066 21939738 10.1016/j.neuroscience.2011.08.066 PMC3357127

[12] Surmeier DJ, Carrillo-Reid L, Bargas J (2011) Dopaminergic modulation of striatal neurons, circuits, and assemblies. Neuroscience 198: 3–18 doi:10.1016/j.neuroscience.2011.08.051 21906660 10.1016/j.neuroscience.2011.08.051 PMC3235731

[13] Hasbi A, O’Dowd BF, George SR (2010) Heteromerization of dopamine D2 receptors with dopamine D1 or D5 receptors generates intracellular calcium signaling by different mechanisms. Curr Opin Pharmacol 10: 93–99 doi:10.1016/j.coph.2009.09.011 19897420 10.1016/j.coph.2009.09.011 PMC2818238

[14] Hasbi A, Fan T, Alijaniaram M, Nguyen T, Perreault ML, et al (2009) Calcium signaling cascade links dopamine D1-D2 receptor heteromer to striatal BDNF production and neuronal growth. Proc Natl Acad Sci USA 106: 21377–21382 doi:10.1073/pnas.0903676106 19948956 10.1073/pnas.0903676106 PMC2795506

[15] Gerfen CR, Engber TM, Mahan LC, Susel Z, Chase TN, et al (1990) D1 and D2 dopamine receptor-regulated gene expression of striatonigral and striatopallidal neurons. Science 250: 1429–1432. 2147780 10.1126/science.2147780

[16] Meador-Woodruff JH, Mansour A, Healy DJ, Kuehn R, Zhou QY, et al (1991) Comparison of the distributions of D1 and D2 dopamine receptor mRNAs in rat brain. Neuropsychopharmacology 5: 231–242. 1839499

[17] Weiner DM, Levey AI, Sunahara RK, Niznik HB, O’Dowd BF, et al (1991) D1 and D2 dopamine receptor mRNA in rat brain. Proc Natl Acad Sci USA 88: 1859–1863. 1825729 10.1073/pnas.88.5.1859 PMC51125

[18] Lester J, Fink S, Aronin N, DiFiglia M (1993) Colocalization of D1 and D2 dopamine receptor mRNAs in striatal neurons. Brain Research 621: 106–110 doi:––10.1016/0006–8993(93)90303–5 8221060 10.1016/0006-8993(93)90303-5

[19] Larson ER, Ariano MA (1994) Dopamine receptor binding on identified striatonigral neurons. Neurosci Lett 172: 101–106. 8084509 10.1016/0304-3940(94)90672-6

[20] Le Moine C, Bloch B (1995) D1 and D2 dopamine receptor gene expression in the rat striatum: sensitive cRNA probes demonstrate prominent segregation of D1 and D2 mRNAs in distinct neuronal populations of the dorsal and ventral striatum. J Comp Neurol 355: 418–426 doi:10.1002/cne.903550308 7636023 10.1002/cne.903550308

[21] Surmeier DJ, Song WJ, Yan Z (1996) Coordinated expression of dopamine receptors in neostriatal medium spiny neurons. J Neurosci 16: 6579–6591. 8815934 10.1523/JNEUROSCI.16-20-06579.1996 PMC6578920

[22] Rashid AJ, So CH, Kong MMC, Furtak T, El-Ghundi M, et al (2007) D1-D2 dopamine receptor heterooligomers with unique pharmacology are coupled to rapid activation of Gq/11 in the striatum. Proc Natl Acad Sci USA 104: 654–659 doi:10.1073/pnas.0604049104 17194762 10.1073/pnas.0604049104 PMC1766439

[23] Wong AC, Shetreat ME, Clarke JO, Rayport S (1999) D1- and D2-like dopamine receptors are co-localized on the presynaptic varicosities of striatal and nucleus accumbens neurons in vitro. Neuroscience 89: 221–233. 10051231 10.1016/s0306-4522(98)00284-x

[24] Aizman O, Brismar H, Uhlén P, Zettergren E, Levey AI, et al (2000) Anatomical and physiological evidence for D1 and D2 dopamine receptor colocalization in neostriatal neurons. Nat Neurosci 3: 226–230 doi:10.1038/72929 10700253 10.1038/72929

[25] Lee SP, So CH, Rashid AJ, Varghese G, Cheng R, et al (2004) Dopamine D1 and D2 receptor Co-activation generates a novel phospholipase C-mediated calcium signal. J Biol Chem 279: 35671–35678 doi:10.1074/jbc.M401923200 15159403 10.1074/jbc.M401923200

[26] Perreault ML, Hasbi A, Alijaniaram M, O’Dowd BF, George SR (2012) Reduced striatal dopamine D1-D2 receptor heteromer expression and behavioural subsensitivity in juvenile rats. Neuroscience 225: 130–139 doi:10.1016/j.neuroscience.2012.08.042 22986162 10.1016/j.neuroscience.2012.08.042 PMC3479309

[27] Valjent E, Bertran-Gonzalez J, Hervé D, Fisone G, Girault J-A (2009) Looking BAC at striatal signaling: cell-specific analysis in new transgenic mice. Trends Neurosci 32: 538–547 doi:10.1016/j.tins.2009.06.005 19765834 10.1016/j.tins.2009.06.005

[28] Chan CS, Peterson JD, Gertler TS, Glajch KE, Quintana RE, et al (2012) Strain-Specific Regulation of Striatal Phenotype in Drd2-eGFP BAC Transgenic Mice. The Journal of neuroscience: the official journal of the Society for Neuroscience 32: 9124–9132 doi:–10.1523/JNEUROSCI.0229–12.2012 22764222 10.1523/JNEUROSCI.0229-12.2012 PMC3461272

[29] Nelson AB, Hang GB, Grueter BA, Pascoli V, Luscher C, et al (2012) A Comparison of Striatal-Dependent Behaviors in Wild-Type and Hemizygous Drd1a and Drd2 BAC Transgenic Mice. The Journal of neuroscience: the official journal of the Society for Neuroscience 32: 9119–9123 doi:–10.1523/JNEUROSCI.0224–12.2012 22764221 10.1523/JNEUROSCI.0224-12.2012 PMC3420343

[30] Shuen JA, Chen M, Gloss B, Calakos N (2008) Drd1a-tdTomato BAC transgenic mice for simultaneous visualization of medium spiny neurons in the direct and indirect pathways of the basal ganglia. J Neurosci 28: 2681–2685 doi:–10.1523/JNEUROSCI.5492–07.2008 18337395 10.1523/JNEUROSCI.5492-07.2008 PMC6670676

[31] Gong S, Zheng C, Doughty ML, Losos K, Didkovsky N, et al (2003) A gene expression atlas of the central nervous system based on bacterial artificial chromosomes. Nature 425: 917–925 doi:10.1038/nature02033 14586460 10.1038/nature02033

[32] Mendez JA, Bourque M-J, Dal Bo G, Bourdeau ML, Danik M, et al (2008) Developmental and target-dependent regulation of vesicular glutamate transporter expression by dopamine neurons. J Neurosci 28: 6309–6318 doi:–10.1523/JNEUROSCI.1331–08.2008 18562601 10.1523/JNEUROSCI.1331-08.2008 PMC6670902

[33] Fasano C, Thibault D, Trudeau L-E (2008) Culture of postnatal mesencephalic dopamine neurons on an astrocyte monolayer. Curr Protoc Neurosci Chapter 3: Unit 3.21. doi:10.1002/0471142301.ns0321s44. 10.1002/0471142301.ns0321s44 18633997

[34] Fortin GM, Bourque M-J, Mendez JA, Leo D, Nordenankar K, et al (2012) Glutamate corelease promotes growth and survival of midbrain dopamine neurons. J Neurosci 32: 17477–17491 doi:–10.1523/JNEUROSCI.1939–12.2012 23197738 10.1523/JNEUROSCI.1939-12.2012 PMC6621856

[35] Shetreat ME, Lin L, Wong AC, Rayport S (1996) Visualization of D1 dopamine receptors on living nucleus accumbens neurons and their colocalization with D2 receptors. J Neurochem 66: 1475–1482. 8627301 10.1046/j.1471-4159.1996.66041475.x

[36] Ade KK, Wan Y, Chen M, Gloss B, Calakos N (2011) An Improved BAC Transgenic Fluorescent Reporter Line for Sensitive and Specific Identification of Striatonigral Medium Spiny Neurons. Front Syst Neurosci 5: 32 doi:10.3389/fnsys.2011.00032 21713123 10.3389/fnsys.2011.00032 PMC3113108

[37] Araki KY, Sims JR, Bhide PG (2007) Dopamine receptor mRNA and protein expression in the mouse corpus striatum and cerebral cortex during pre- and postnatal development. Brain Res 1156: 31–45 doi:10.1016/j.brainres.2007.04.043 17509542 10.1016/j.brainres.2007.04.043 PMC1994791

[38] Specht LA, Pickel VM, Joh TH, Reis DJ (1981) Light-microscopic immunocytochemical localization of tyrosine hydroxylase in prenatal rat brain. II. Late ontogeny. J Comp Neurol 199: 255–276 doi:10.1002/cne.901990208 6114115 10.1002/cne.901990208

[39] Voorn P, Kalsbeek A, Jorritsma-Byham B, Groenewegen HJ (1988) The pre- and postnatal development of the dopaminergic cell groups in the ventral mesencephalon and the dopaminergic innervation of the striatum of the rat. Neuroscience 25: 857–887. 3405431 10.1016/0306-4522(88)90041-3

[40] Perrone-Capano C, Di Porzio U (2000) Genetic and epigenetic control of midbrain dopaminergic neuron development. Int J Dev Biol 44: 679–687. 11061432

[41] Ferrari DC, Mdzomba BJ, Dehorter N, Lopez C, Michel FJ, et al (2012) Midbrain dopaminergic neurons generate calcium and sodium currents and release dopamine in the striatum of pups. Front Cell Neurosci 6: 7 doi:10.3389/fncel.2012.00007 22408606 10.3389/fncel.2012.00007 PMC3297358

[42] Swift JL, Godin AG, Doré K, Freland L, Bouchard N, et al (2011) Quantification of receptor tyrosine kinase transactivation through direct dimerization and surface density measurements in single cells. Proc Natl Acad Sci USA 108: 7016–7021 doi:10.1073/pnas.1018280108 21482778 10.1073/pnas.1018280108 PMC3084083

[43] Le Moine C, Normand E, Bloch B (1991) Phenotypical characterization of the rat striatal neurons expressing the D1 dopamine receptor gene. Proc Natl Acad Sci USA 88: 4205–4209. 1827915 10.1073/pnas.88.10.4205 PMC51627

[44] Paxinos G, Watson C (2007) Atlas of the Developing Mouse Brain: At E17.5, PO, and. Academic Press. 378 p.

[45] Paxinos G, Franklin KBJ (2004) The Mouse Brain in Stereotaxic Coordinates. Gulf Professional Publishing. 138 p.

